# Troxerutin, a herbal metabolite with antidiabetic and antihypercholesterolemic potential, regulates metabolic gene activity in male diabetic rats

**DOI:** 10.3389/fphar.2025.1687575

**Published:** 2026-01-28

**Authors:** Saira Gul, Mahrukh Naseem, Irfan Shahzad Sheikh, Hafiz Muhammad Ali, Imtiaz Rabbani, Tariq Jamil, Sehar Gul, Zaid Chachar, Sana Ullah, Saima Asif, Farid Shokry Ataya, Dalia Fouad, Kasim Sakran Abass, Yuanzhe Cai, Jieren Liu, Feijuan Huang

**Affiliations:** 1 Department of Zoology, University of Balochistan, Quetta, Pakistan; 2 Center for Advanced Studies in Vaccinology and Biotechnology, University of Balochistan, Quetta, Pakistan; 3 Faculty of Veterinary and Animal Sciences, The Islamia University of Bahawalpur, Bahawalpur, Pakistan; 4 Department of Physiology, University of Veterinary and Animal Sciences, Lahore, Pakistan; 5 Independent Researcher, Jena, Germany; 6 Department of Microbiology, University of Balochistan, Quetta, Pakistan; 7 Department of Data Science, The Chinese University of Hong Kong, Shenzhen, China; 8 Institute of Molecular Biology and Biotechnology, Bahauddin Zakariya University, Multan, Pakistan; 9 Department of Biochemistry, College of Science, King Saud University, Riyadh, Saudi Arabia; 10 Department of Zoology, College of Science, King Saud University, Riyadh, Saudi Arabia; 11 Department of Physiology, Biochemistry and Pharmacology, College of Veterinary Medicine, University of Kirkuk, Kirkuk, Iraq; 12 Shenzhen Technology University, Shenzhen, China; 13 Shenzhen Second People’s Hospital, Shenzhen, China

**Keywords:** lipid metabolic genes, lipid profile, metformin, mRNA expression, rats, troxerutin, type 2 diabetes

## Abstract

**Background/Objective:**

This study evaluated the antidiabetic and antihypercholesterolemic potential of the botanical metabolite troxerutin (TRX) and compared it with that of metformin in high-fat diet-fed streptozotocin-induced diabetic male Wistar rats.

**Methods:**

The rats (n = 48) were divided into six groups. Diabetes was induced in the treatment groups, and different doses of troxerutin (TRX)—25 mg/kg/day (TRX25-D), 50 mg/kg/day (TRX50-D), and 75 mg/kg/day (TRX75-D)—or the standard drug (10 mg/kg/day; MET10-D) were administered for a period of 7 weeks, compared to the negative (non-diabetic control, NDC) and positive (diabetic control, DC) control groups. At the end of the trial period, serum was collected to determine the lipid profile (high-density lipoprotein, low-density lipoprotein, and very-low-density lipoprotein (VLDL)) and the concentrations of hepatic (aspartate aminotransferase and alanine aminotransferase), renal (urea and creatinine), and oxidative stress (catalase and malondialdehyde) markers. Adipose tissue, skeletal muscle, and liver tissue samples were collected to determine mRNA expression, of pro-inflammatory cytokines [tumor necrosis factor-α (TNF-α) and interleukin-6 (IL-6)] and genes involved in lipid metabolism [peroxisome proliferator-activated receptor α (PPARα), peroxisome proliferator-activated receptor γ (PPARγ), fatty acid synthase (FAS), and sterol regulatory element-binding protein-1c (SREBP-1c)].

**Results:**

The results showed a significant decrease (p < 0.05) in total cholesterol (TC), triglycerides (TGs), VLDL, and LDL levels, along with hepatic, renal, and stress markers, in the rats treated with a higher concentration of troxerutin (TRX75-D) compared to diabetic control rats. Moreover, troxerutin significantly (p < 0.05) upregulated the expression of PPARα and PPARγ, while the expression of FAS, SREBP-1c, TNF-α, and IL-6 genes were significantly (p < 0.05) downregulated simultaneously in the adipose tissue, skeletal muscles, and liver in a dose-dependent manner, compared to diabetic ct control rats.

**Conclusion:**

Troxerutin has considerable antidiabetic and antihypercholesterolemic potential and thus could be safely used as an alternative therapeutic compound to the standard antidiabetic drug metformin.

## 1 Introduction

Diabetes mellitus (DM) is a chronic metabolic disorder characterized by persistent hyperglycemia due to impaired insulin secretion (Ojo et al., 2023). Type-II diabetes mellitus (T2DM) accounts for nearly 90% of diabetic cases worldwide (Reed et al., 2021; Yameny, 2024). According to the International Diabetes Federation (IDF), more than 537 million adults are currently living with diabetes, and an additional 240 million remain undiagnosed, contributing to annual global healthcare costs approaching USD 966 billion (Hossain et al., 2024). The prevalence has increased from 4.6% (151 million) in 2000 to 10.5% (537 million) in 2021 and is projected to reach 12.2% (783 million) by 2045 (Hossain et al., 2024). Pakistan ranks second globally, after China and India, in the prevalence of diabetes (Azeem et al., 2022) and 10th in obesity (Tanveer et al., 2022).

T2DM is the most prevalent form of diabetes, characterized by insulin resistance, pancreatic β-cell dysfunction, and metabolic imbalance. Type-II diabetes is closely associated with obesity as the majority of these patients are overweight or obese. In particular, visceral fat contributes to increased deposition of intra-hepatic and intra-muscular triglycerides, exacerbating insulin resistance and pancreatic β-cell dysfunction (Klein et al., 2022). Obesity is the most significant risk factor for development and progression of T2DM, and obese men and women have a 7- and 12-fold increased risk of developing the disease, respectively (Chandrasekaran and Weiskirchen, 2024). Hence, due to co-existence of metabolic disorders and their inter-connected patho-physiological effects, the term “diabesity” is used to demonstrate the strong interlink between obesity and diabetes (Michaelidou et al., 2023). Approximately, 80% of T2DM patients present with central obesity, emphasizing adiposity as a key contributing factor to of this disease (Russo and Lumeng, 2018; Wondmkun, 2020). Additional risk factors include genetic predisposition, advanced age (predominantly >40 years), and associated lifestyle contributors such as a diet rich in synthetic sugars, saturated fatty acids, and low fiber; physical inactivity; and the use of tobacco, statins, glucocorticoids, diuretics, thiazide, and antipsychotics (Korat et al., 2014; Hope et al., 2016). Hence, obesity is equally associated with an increased risk of T2DM, hypertension, cardiovascular disease, and dyslipidemia (Yamada et al., 2023). Dyslipidemia is a key determinant of T2DM, typically presenting as elevated triglycerides (TGs) and low-density lipoproteins (LDLs) with reduced high-density lipoproteins (HDLs), which together increase the risk of atherosclerosis, cardiovascular events, and metabolic inflammation (Berisha et al., 2025). Thus, hyper- and hypolipidemia lead to endocrine disturbances, hepatic dysfunction, and broader metabolic complications (Chatterjee et al., 2017).

Metformin is a first-line antidiabetic drug that reduces hepatic gluconeogenesis, improves peripheral insulin sensitivity, and facilitates glucose uptake without inducing hypoglycemia (Cwynar-Zając, 2021). It is used to treat obesity, gestational diabetes, and polycystic ovarian syndrome (Gonzalez-Lopez and Wojeck, 2023). However, its clinical utility induces gastro-intestinal discomfort, vitamin B12 deficiency, and the risk of lactic acidosis (Polyakova et al., 2022; Sanchez-Rangel and Inzucchi, 2017). Moreover, dyslipidemia in T2DM patients increases the risk of cardiovascular disease, underscoring the need for developing safer and more effective alternatives (Bagnati et al., 2021). Hence, despite the availability of conventional drugs such as metformin and sulfonylureas, their adverse effects have prompted the exploration of safer, plant-derived alternatives to these drugs. Considering the side effects of chemical drugs, attention has been directed toward phyto-therapeutics as complementary or alternative interventions (Aslam et al., 2023a; Aslam et al., 2023b; Aslam et al., 2025).

Troxerutin is a naturally occurring water-soluble bioflavonoid metabolite of rutin (rutoside; vitamin P4), primarily derived from the flowers of *Styphnolobium japonicum* (L.) Schott (Fabaceae) and also present in various botanicals such as tea, coffee, vegetables, and fruits (Ahmadi et al., 2021; Masood et al., 2020). Troxerutin is a potent antioxidant, anti-inflammatory, and vaso-protective agent that can control hyperlipidemia, oxidative stress, and capillary permeability (Zamanian et al., 2021). It is hydrophilic in nature, is rapidly absorbed by the gastrointestinal tract, and shows efficient anti-hypertriglyceridemic and anti-hypercholesterolemic activities; thus, it is found to be effective in treating cardiovascular disorders (Subbaraj et al., 2023; Lee et al., 2024; Shah et al., 2022; Yu and Zheng, 2017). Despite having potent anti-inflammatory and anti-hyperlipidemic effects, the underlying molecular mechanisms of its activity across multiple tissues remain incompletely understood. Thus, the authors wanted to evaluate the antidiabetic and antihypercholesterolemic potential of troxerutin in experimentally induced diabetic rats and elucidate its potential mechanism of the action and modulation of genetic factors in key insulin-responsive tissues. This study investigated the gene-level regulatory effects of troxerutin on key metabolic and inflammatory genes involved in glycemic and lipid metabolism, offering mechanistic insights relevant to metabolic disease management and elucidating its therapeutic potential as an alternative to metformin, a standard antidiabetic drug. We hypothesized that troxerutin ameliorates the effects of diabetes and dyslipidemia through coordinated regulation of the metabolic and inflammatory gene network by upregulating the expressions of lipid oxidation regulators [peroxisome proliferator-activated receptor α (PPARα) and peroxisome proliferator-activated receptor γ (PPARγ)] and downregulating those of the lipogenic and pro-inflammatory mediators [fatty acid synthase (FAS), sterol regulatory element-binding protein-1c (SREBP-1c), tumor necrosis factor-α (TNF-α), and interleukin-6 (IL-6)], thereby restoring metabolic homeostasis.

## 2 Materials and methods

### 2.1 Animal housing and management

A total of 48 (n = 48) healthy, normoglycemic male Wistar rats (120 days old, with a mean body weight of 218.46 ± 0.90 g) were procured for this study. The animals were maintained under controlled environmental conditions (temperature: 24 °C ± 2 °C, relative humidity: 55%–65%, and light/dark cycle: 12/12 h) in the animal facility of the University of Balochistan, Quetta (Afzal et al., 2024; Feng et al., 2024). All the animals were provided with commercially available standard rat chow (purified rat diet: crude protein = 19%–22%, crude fiber = 3.5%–5%, and 2,650 Kcal/Kg of metabolizable energy) twice daily, with *ad libitum* access to clean filtered drinking water. The rats were acclimatized for 1 week before the start of the experimental trial. Randomization was performed by computer-generated allocation to minimize selection bias. The body weight and fasting blood sugar levels were recorded weekly throughout the experiment.

All experimental protocols were reviewed and approved by the Institutional Animal Ethics Committee of the University of Balochistan, Quetta, Pakistan (Approval No.: UOB/IAEC/2024/17). The study adhered to the ARRIVE guidelines and the National Institutes of Health Guide for the Care and Use of Laboratory Animals (NIH Publication No. 8023, revised 1978).

### 2.2 Chemicals used in the study

Troxerutin (≥98% purity, Cat. No. T2397) and streptozotocin (STZ, ≥98% purity, Cat. No. S0130) were purchased from Sigma-Aldrich (St. Louis, MO, USA). Troxerutin was dissolved freshly in 0.5% carboxymethylcellulose just before administration. Metformin (≥99% purity, Cat. No. M107827, Merck, Germany) was used as the reference standard. The blood glucose level was measured using a glucometer (ACCU-CHEK^®^; Roche Diagnostics, Basel, Switzerland) and validated with spectrophotometric assays. Commercial assay kits for the serum lipid profile including total cholesterol (TC), TGs, HDL, LDL, and very-low-density lipoprotein (VLDL) were procured from Innoline Diagnostic Kits (Martin Dow Specialties Pvt. Ltd., Gien, France; distributed by Medisurg Pvt. Ltd., Karachi, Pakistan). Hepatic [aspartate aminotransferase (AST) and alanine aminotransferase (ALT)] and renal [urea and creatinine (CRT)] biomarker kits were supplied by Randox Laboratories Ltd. (Crumlin, UK). Catalase (CAT) and malondialdehyde (MDA) activity kits were obtained from Cayman Chemical (Ann Arbor, MI, USA).

For molecular analyses, TRIzol™ reagent (Invitrogen™, Thermo Fisher Scientific, USA) was used for RNA extraction, and the RevertAid First Strand cDNA SuperScript™ III Reverse Transcriptase Kit (Thermo Fisher Scientific, USA; Cat. No. K1622) was used for cDNA synthesis. Quantitative PCR was conducted using SYBR™ Green Universal Master Mix (Applied Biosystems™, USA; Cat. No. 4309155). All the reagents and solvents used in this study were of analytical research grade.

### 2.3 Botanical drug identification

The botanical source of troxerutin was taxonomically validated using *Plants of the World Online* (POWO, http://www.plantsoftheworldonline.org/). Troxerutin is obtained from the flowers of *Styphnolobium japonicum* (L.) Schott (family: Fabaceae), commonly known as the Japanese pagoda tree. The species name and authority were confirmed based on the current botanical nomenclature standards. According to the Chinese Pharmacopoeia and other pharmacopeial references, troxerutin is recognized as a botanical drug with documented therapeutic applications in vascular and metabolic disorders (Bianchi et al., 2018; Malinska et al., 2019).

### 2.4 Induction of diabetes and experimental design

All rats (n = 48) were initially fed a high-fat, moderate-protein, and low-carbohydrate diet formulated to induce insulin resistance prior to streptozotocin administration. The diet consisted of 4.6% cooking oil, 7.5% fructose, 36.3% beef tallow, 12.7% starch, and 39.2% casein (percent by weight). Based on the proximate composition analysis, this formulation provided approximately 45% of total calories from lipids, 35% from protein, and 20% from carbohydrates, yielding an energy density of 4.5 kcal/g. Although the protein contents were higher than those in the standard chow (typically 18%–20%), the inclusion of beef tallow and cooking oil contributed more than two-fold higher fat energy than that in normal control diets (∼10–15% kcal from fat). Similar high-fat diet compositions have been previously used in diet-induced insulin resistance models in rats (Malinska et al., 2019) and mice (Geetha et al., 2017). Thus, although being protein-enriched, the formulation is a lipid-rich diet and is sufficient to promote metabolic dysregulation consistent with early-stage type-II diabetes induction.

Diabetes was induced in the animals followed by overnight fasting by using a single intra-peritoneal dose of streptozotocin (35 mg/kg body weight). To prevent hypoglycemia, a 20% glucose solution was administered orally for the first 6 h post-injection, followed by 5% glucose solution in drinking water for the next 24 h. The blood glucose levels were measured from the blood of the tail vein by using a glucometer ACCU-CHEK^®^. The rats with blood glucose levels ≥250 mg/dL at 72 h after streptozotocin administration were considered diabetic and included in this study, according to the earlier procedure (Srinivasan et al., 2018). The rats were randomly divided into six groups (n = 8 rats/group; as described underneath in Section 2.6): a non-diabetic control group (NDC; negative control group) consisting of healthy rats without any treatment, receiving only normal saline solution (0.5 mL) intraperitoneally; a diabetic control group (DC; positive control group) consisting of diabetic rats without any treatment; a metformin-treated diabetic group (MET10-D), in which the diabetic rats received metformin (10 mg/kg/day); and three troxerutin-treated diabetic groups receiving troxerutin administered orally at the doses of 25 mg/kg/day (TRX25-D), 50 mg/kg/day (TRX50-D), or 75 mg/kg/day (TRX75-D) for a period of 7 weeks. The behavioral alterations were continuously observed in the treated mice throughout the experiment. At the end of the trial, the rats were euthanized by controlled CO_2_ inhalation in accordance to the approved ethical guidelines, and all efforts were made to minimize animal suffering (Ali et al., 2014b; Urbinati et al., 2016).

### 2.5 Dose selection and treatment duration

TRX was tested at concentrations of 25, 50, or 75 mg·/kg/·day to span a sub-to-mid-preclinical range while permitting dose–response analyses on metabolic and inflammatory gene expression. Previous rodent studies have demonstrated the efficacy of TRX at 50 mg–150 mg/kg/day across metabolic phenotypes, including improvement in lipid management and insulin sensitivity at 150 mg/kg/day for 4 weeks in a non-obese rat model of metabolic hereditary hypertriglyceridemic syndrome (Malinska et al., 2019) and cardio-metabolic benefits at 150 mg/kg/day in high-fat and high-fructose-fed mice for 60 days (Geetha et al., 2014). These reports substantiate our upper dose limit (75 mg/kg) as a conservative fraction of commonly used effective levels, while enabling graded molecular responses to be resolved in liver, muscle, and adipose tissues. Metformin was administered at 10 mg·/kg/·day as a low-dose positive control to benchmark the directionality of effects without fully normalizing glycemia/lipids, which could mask TRX-specific transcriptional changes. We have previously noted that metformin was administered up-to 200 mg–300 mg/kg/day for 8 weeks to maximize the glycemic efficacy in high-fat-fed streptozotocin-induced type 2 diabetic Wistar rats (Zhou et al., 2024). We intentionally chose a lower dose to minimize the ceiling effects and facilitate the detection of TRX-driven gene regulations; accordingly, we avoid any claims of statistical equivalence to metformin elsewhere in the manuscript. Moreover, our 7-week treatment period aligns with prior chronic TRX protocols (8–20 weeks) that capture stable hepatic and peripheral adaptations in lipid metabolism and oxidative stress pathways (Liu et al., 2010; Lu et al., 2011; Zhang et al., 2014; Zhang et al., 2016), while fitting our HFD/STZ timeline for induction and stabilization of the diabetic phenotype.

### 2.6 Randomization and blinding

The animals were randomly allocated to experimental groups using a computer-generated block randomization list (blocks of 6–8) prepared by an investigator not involved in dosing or outcome assessment. The allocation was concealed with sequentially numbered, opaque and sealed envelopes opened only at the time of first dosing. The cages were balanced to avoid cage effects. i.e., no more than two animals/group/rack level and rotated on a weekly basis. The personnel administering the dosage were distinct from the outcome assessors. The serum and tissues sample tubes were labeled with anonymized codes so that laboratory staff performing the biochemical assays and qPCR were blinded to group assignments, until the completion of primary analyses. The data analysts remained blinded during data cleaning and initial statistics; unblinding was done only after the analysis plan was executed and figures/tables were finalized. Moreover, to minimize measurement bias, the biochemistry plate layouts and qPCR run orders were randomized across the groups, with interplate calibrators and no-template controls included according to the Minimum Information for Publication of Quantitative Real-Time PCR Experiments (MIQE) recommendations (Bustin et al., 2009).

### 2.7 Determination of the blood glucose and serum biochemical profiles

The fasting blood glucose level was measured after 12 h of overnight fasting using a glucose meter (ACCU-CHEK^®^). The blood samples were collected in EDTA-free tubes, and serum was separated by centrifugation at 5,000 rpm for 5 min and then stored at −20 °C until analysis (Ayub et al., 2018; Malik et al., 2018; Qureshi and Ali, 2016). The serum profiles of TC, TGs, HDL, VLDL, and LDL were analyzed using commercially available kits (Innoline, Martin Dow Specialties (Pvt.) Ltd., Gien, France). The hepato-renal function markers, including AST (IU/L), ALT (IU/L), urea (mg/dL), and CRT (mg/dL), were measured using commercially available biochemical kits (Al-Farraj et al., 2024). The oxidative stress parameters were assessed by quantifying CAT (Ku/L) (Mahmood et al., 2024; Naz et al., 2025) and MDA (mmol/L) levels using a Shimadzu 1700 UV-visible spectrophotometer at 240 nm and 720 nm, respectively.

### 2.8 Gene expression analysis

The skeletal muscle, liver, and adipose tissue samples were collected from the rats immediately after euthanasia and stored at −80 °C until further analysis (Rasheed et al., 2024; Sikandar et al., 2020). The total RNA was extracted using TRIzol™ reagent according to the manufacturer’s protocol. RNA concentration was determined at 260 nm using a spectrophotometer, and the purity was assessed using A260/A280 and A260/A230 ratios that consistently ranged from 1.9 to 2.1 and from 2.0 to 2.3, respectively, indicating high-quality RNA, free from protein or phenol contamination. RNA integrity was further confirmed by agarose gel electrophoresis showing distinct 28S and 18S rRNA bands.

The synthesis of complementary DNA (cDNA) and quantitative PCR were then conducted by following the MIQE guidelines, to ensure reproducibility. The cDNA was synthesized from 1 µg of total RNA, and quantitative real-time PCR (RT-qPCR) was performed under optimized cycling conditions: 95 °C for 10 min; 40 cycles of 95 °C for 15 s and 60 °C for 1 min (Ali et al., 2014a; 2014b). The primers for target genes including PPARα, PPARγ, FAS, SREBP-1c, TNF-α, and IL-6 were designed using the Primer3 web tool (Table 1). The primer efficiencies were validated using five-point serial dilution standard curves, yielding efficiencies ranging between 95% and 105% (*R* ^2^ ≥ 0.99). All reactions were performed in triplicate, including no-template and no-reverse-transcription controls to confirm the absence of contamination and genomic DNA. Relative gene expression levels were calculated using the comparative 2^–ΔΔCt^ method (Dupain et al., 2016; Urbinati et al., 2016), normalized to the housekeeping gene glyceraldehyde-3-phosphate dehydrogenase (GAPDH) and expressed as fold changes relative to the control group.

**TABLE 1:** Primer sequences of lipid metabolic genes used in the study.

Gene	Forward primer (5′-3′)	Reverse primer (5′-3′)
PPARα	GAG​ACC​CTC​GGG​GAT​CTT​AG	CGT​CTT​GTG​TCC​TGA​GCT​TG
PPARγ	CTG​ACC​CAA​TGG​TTG​CTG​ATT​AC	GGA​CGC​AGG​CTC​TAC​TTT​GAT​C
FAS	CGC​CGT​GGT​GCT​GGA​GAT​TG	CTT​GCC​GAG​GTT​GGT​GAG​GAA​G
SREBP-1c	GGC​ATG​AAA​CCT​GAA​GTG​GT	TGG​GCT​TTC​ACC​TGG​TTA​TC
TNFα	GCA​GAG​CCT​TCC​AAG​CCT​ACC	GTT​ACC​CAG​CCC​ACC​TCC​TTT​G
IL-6	AGT​CCG​GAG​AGG​AGA​CTT​CA	TTG​CCA​TTG​CAC​AAC​TCT​TT
GAPDH	TCC​CAT​TCT​TCC​ACC​TTT​GAT​GCT	ACC​CTG​TTG​CTG​TAG​CCA​TAT​TCA​T

GAPDH, glyceraldehyde-3-phosphate dehydrogenase; PPARα, peroxisome proliferator-activated receptor α; PPARγ, peroxisome proliferator-activated receptor γ; FAS, fatty acid synthase; SREBP-1c, sterol regulatory element-binding protein-1c; TNF-α, tumor necrosis factor α; IL-6, interleukin-6.

### 2.9 Statistical analysis

All data are expressed as mean ± standard error of the mean (SEM) based on n = 8 biological replicates/group, with each experiment performed in triplicate. All statistical analyses were performed using one-way analysis of variance (ANOVA) followed by Tukey’s multiple comparison post hoc test (IBM SPSS Statistics v25.0, IBM Corp., Armonk, NY, USA). Where the data did not meet the assumptions of normality and equal variance, a nonparametric Kruskal–Wallis test followed by Dunn’s post hoc correction was applied. The statistical significance was denoted as p < 0.05. The exact *p*-values were computed and reported for all group comparisons to ensure statistical transparency. However, extremely small *p*-values (*p* < 0.001) were reported as such. The figures and tables are updated accordingly to precise significance levels, where p < 0.05 = *, p < 0.01 = **, and p < 0.001 = ***.

## 3 Result

### 3.1 Serum lipid profile

The DC group showed a significant increase (p < 0.01) in serum total cholesterol and triglycerides compared to the NDC group. Treatment with troxerutin (TRX25-D, TRX50-D, and TRX75-D) significantly reduced the serum TC and TG levels in a dose-dependent manner relative to the DC rats (p < 0.05; Table 2). The values of HDL were significantly (p < 0.05) reduced in the diabetic (DC) rats, but troxerutin treatment (TRX25-D; p = 0.041, TRX50-D; p = 0.018, and TRX75-D; p = 0.007) significantly increased the HDL levels in the treated groups compared to the DC group. Although LDL and VLDL levels were elevated in the DC rats, they were significantly reduced following troxerutin treatment (p = 0.028 and p = 0.014, respectively). The MET10-D group also demonstrated comparable effects on HDL (p = 0.012) and LDL (p = 0.009) compared to DC rats. Hence, troxerutin therapy significantly increased the HDL level and significantly reduced the contents of VLDL and LDL in the serum of treated diabetic animals in a dose-dependent manner, i.e., with a higher dose of troxerutin, demonstrating to be more effective (Table 2).

**TABLE 2:** Effects of different concentrations of troxerutin on the serum lipid profile (mg/dL).

Group	TC	TG	HDL	LDL	VLDL
NDC	86.13 ± 3.11^c^	73.13 ± 1.75^d^	52.88 ± 1.48^a^	18.63 ± 1.14^d^	14.63 ± 0.35^d^
DC	168.50 ± 11.69^a^	136.25 ± 2.17^a^	30.13 ± 0.99^e^	111.13 ± 12.60^a^	27.25 ± 0.43^a^
MET10-D	125.75 ± 4.78^b^	109.38 ± 2.99^b^	40.13 ± 1.14^d^	63.75 ± 2.48^bc^	21.88 ± 0.60^b^
TRX25-D	136.82 ± 3.53^b^	105.63 ± 2.56^bc^	42.38 ± 1.34^cd^	73.25 ± 3.93^b^	21.13 ± 0.51^bc^
TRX50-D	129.25 ± 3.13^b^	102.38 ± 1.67^c^	45.13 ± 1.98^bc^	63.65 ± 1.77^bc^	20.48 ± 0.33^c^
TRX75-D	123.16 ± 2.95^b^	101.22 ± 1.37^c^	49.13 ± 1.60^ab^	53.63 ± 2.50^c^	20.25 ± 0.27^c^

Troxerutin treatment significantly (p < 0.05) reduced the TC, and TG, levels in the serum of treated rats compared to animals of the DC group. The HDL values were significantly (p < 0.05) increased, while the VLDL and LDL levels were significantly (p < 0.05) reduced, with an increase in the dose of troxerutin in the treated diabetic rats. Data are presented as mean ± SEM (n = 8/group).

The different superscripts (a–e) in a column show the statistical difference among various experimental groups. Statistical tests: one-way ANOVA followed by Tukey’s post hoc test (applied to normally distributed data) and the Kruskal–Wallis test with Dunn’s correction (applied to non-parametric data).

TC, total cholesterol; TG, total triglyceride; HDL, high-density lipoprotein; VLDL, very low-density lipoprotein; LDL, low-density lipoprotein; MET, metformin; TRX, troxerutin.

### 3.2 Body weight and blood glucose level

Throughout the treatment period of 7-weeks, the DC rats exhibited progressive weight loss (−17.5% ± 1.3%) and sustained hyperglycemia (>320 mg/dL), indicating severe insulin resistance and catabolic stress. Troxerutin produced a dose-dependent attenuation of these effects: TRX25-D and TRX50-D groups showed partial recovery, while TRX75-D restored the body weight to 92% ± 4% of baseline data and reduced the fasting glucose level to 146 ± 12 mg/dL, closely approximating that of the metformin group (MET10-D: 138 ± 9 mg/dL). Moreover, serum insulin levels were increased modestly but significantly in TRX50-D and TRX75-D (p < 0.05) groups, suggesting improved β-cell function and insulin sensitivity rather than hyperinsulinemia.

### 3.3 Hepato-renal and oxidative stress markers

The DC group exhibited marked hepatic and renal dysfunction relative to the NDC group, with serum AST, ALT, CRT, and urea levels elevated by +62%, +71%, +58%, and +64%, respectively (all p < 0.001). Troxerutin treatment led to a significant improvement in these parameters in a dose-dependent manner when compared to DC rats. AST decreased by −23% (TRX25-D, p = 0.041), −37% (TRX50-D, p = 0.018), and −51% (TRX75-D, p = 0.006), while ALT decreased by −21%, −35%, and −48%, respectively. Renal markers showed similar trends, with urea reduced by −33% (TRX75-D, p = 0.008) and CRT by −39% (TRX75-D, p = 0.005) compared to DC. Metformin treatment (MET10-D) produced comparable improvements (AST: −47% and ALT: −45%, p < 0.01 vs. DC). In parallel, the oxidative stress parameters showed substantial improvement. The diabetic rats showed elevated hepatic MDA levels (+89% vs. NDC, p < 0.001) and decreased CAT activity (−52% vs. NDC, p < 0.001). Troxerutin treatment decreased MDA levels up to −46% (TRX75-D, p = 0.009) and increased CAT activity by +59% (TRX75-D, p = 0.007) dose-dependently relative to DC animals, demonstrating potent antioxidant restoration. The highest dose of troxerutin (TRX75-D) restored hepatic, renal, and oxidative indices to levels statistically indistinguishable from those of NDC (all p > 0.05), confirming a near-complete functional recovery (Table 3).

**TABLE 3:** Effects of different concentrations of troxerutin on hepatic, renal, and oxidative stress markers.

Group	AST	ALT	Urea	CRT	CAT	MDA
NDC	74.52 ± 0.68^e^	37.12 ± 0.47d^e^	32.71 ± 1.02^f^	1.69 ± 0.13^b^	26.49 ± 0.38^a^	6.26 ± 0.24^b^
DC	165.05 ± 1.26^a^	55.98 ± 0.54^a^	83.27 ± 0.66^a^	2.22 ± 0.16^a^	22.34 ± 0.42^c^	8.41 ± 0.25^a^
MET10-D	76.65 ± 0.49^e^	38.40 ± 0.33^d^	36.12 ± 0.6^e^	1.73 ± 0.12^b^	24.73 ± 0.57^b^	6.37 ± 0.27^b^
TRX25-D	94.21 ± 0.48^b^	47.09 ± 1.41^b^	55.55 ± 0.42^b^	1.79 ± 0.14^b^	20.48 ± 0.39^d^	7.91 ± 0.28^a^
TRX50-D	88.45 ± 1.16^c^	42.01 ± 0.39^c^	47.11 ± 0.56^c^	1.77 ± 0.09^b^	21.20 ± 0.44^cd^	6.52 ± 0.26^b^
TRX75-D	80.52 ± 0.42^d^	36.06 ± 0.73^e^	41.19 ± 0.43^d^	1.76 ± 0.13^b^	23.98 ± 0.56^b^	6.41 ± 0.34^b^

The diabetic rats treated with different concentrations of troxerutin showed a significant (p < 0.05) decrease in hepatic (AST and ALT) and renal (urea and CRT) biomarkers compared to diabetic control rats. Troxerutin treatment significantly (p < 0.05) increased the CAT, activity while significantly (p < 0.05) decreasing the MDA level in the treated diabetic rats compared to the control positive animals, indicating an attenuation in oxidative stress. Data are presented as mean ± SEM (n = 8/group). p < 0.05 was considered statistically significant.

The different superscripts (a-e) in a column show the statistically significance of a parameter among various experimental groups. Statistical tests: one-way ANOVA followed by Tukey’s post hoc test (applied to normally distributed data) and the Kruskal–Wallis test with Dunn’s correction (applied to non-parametric data).

ALT, alanine aminotransferase; AST, aspartate aminotransferase; CRT, creatinine; MDA, malondialdehyde; CAT, catalase; MET, metformin; TRX, troxerutin.

### 3.4 PPARγ and FAS mRNA gene expressions in adipose tissue

**FIGURE 1:**
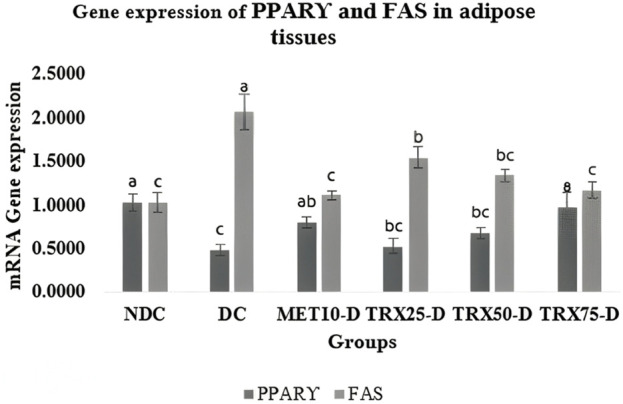
Effects of different concentrations of troxerutin on mRNA expressions of PPARγ and FAS in the adipose tissue of rats. There was a significant (p < 0.05) decrease in PPARγ expression, along with a significant (p < 0.05) upregulation of FAS gene expression, in the adipose tissue of DC rats compared to NDC. Troxerutin treatment significantly (p < 0.05) upregulated PPARγ expression with TRX-75 treatment, while a significant (p < 0.05) downregulation in FAS expression was observed in the treated rats in a dose-dependent manner compared to the animals of the control positive (DC) group. Data are presented as mean ± SEM (n = 8/group). The statistically significant level was set as p < 0.05. The significant difference among various treatment groups has been represented by different superscripts (a–c) mentioned on each bar. Statistical tests: one-way ANOVA followed by Tukey’s post hoc test (applied to normally distributed data) and the Kruskal–Wallis test with Dunn’s correction (applied to non-parametric data). TRX, troxerutin; MET, metformin; NDC, non-diabetic control; DC, diabetic control.

A significant (p < 0.05) down-regulation in the gene expression of PPARγ was observed in the adipose tissue of DC rats compared to NDC rats. Troxerutin significantly (p < 0.05) upregulated PPARγ expression, with more pronounced effects observed in the TRX-75-treated group than in the TRX-25 andTRX-50 groups, as compared with the DC group (Figure 1). FAS gene expression was significantly (p < 0.05) elevated in the DC rats but was significantly (p < 0.05) downregulated in the rats treated with troxerutin in a dose-dependent manner (TRX-25, TRX-50, and TRX-75) compared to the animals of the control positive (DC) group. The highest dose of troxerutin (TRX-75) demonstrated similar alterations in the gene expression results to that of the standard drug, MET-10 (Figure 1).

### 3.5 PPARα mRNA gene expression in skeletal muscles

**FIGURE 2:**
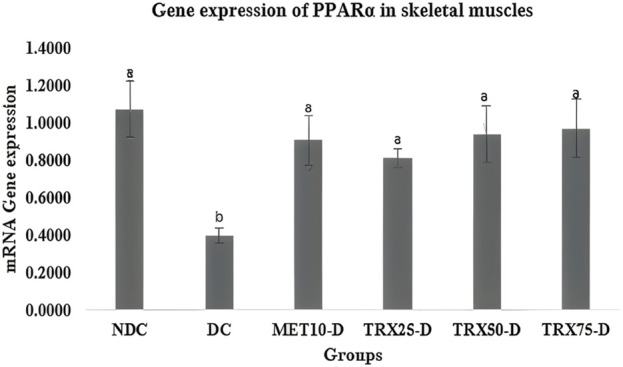
Effects of different concentrations of troxerutin on mRNA gene expressions of PPARα in the skeletal muscles of rats. A significant (p < 0.05) decrease in PPARα gene expression was observed in the skeletal muscles of DC rats compared to NDC, while a significant (p < 0.05) upregulation was observed in the rats treated with different concentrations of troxerutin or with MET-10 compared to the rats of the positive control (DC) group. Different superscripts (a or b) mentioned on each bar show significant differences among various experimental groups. Data are presented as mean ± SEM (n = 8/group). p < 0.05 was considered stastically significant. Statistical tests: one-way ANOVA followed by Tukey’s post hoc test (applied to normally distributed data) and the Kruskal–Wallis test with Dunn’s correction (applied to non-parametric data). TRX, troxerutin; MET, metformin; NDC, non-diabetic control; DC, diabetic control.

PPARα expression in skeletal muscles was significantly reduced (p < 0.05) in the DC rats compared to the non-diabetic control group. Troxerutin treatment produced a dose-dependent increase in PPARα expression, with all three doses (TRX25-D, TRX50-D, and TRX75-D) significantly upregulating its levels relative to the DC group (p < 0.05). The response was comparable to that observed in the metformin-treated group (MET10-D) (Figure 2).

### 3.6 Hepatic PPARα, FAS, and SREBP-1c mRNA expressions

**FIGURE 3:**
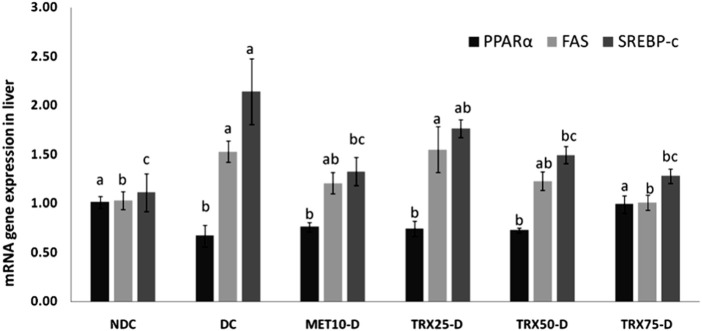
Effects of metformin and different concentrations of troxerutin on mRNA gene expressions of PPARα, FAS, and SREBP-1c in the liver of rats. A significant (p < 0.05) increase in PPARα expression, along with a significant (p < 0.05) downregulation in FAS and SREBP-1c gene expressions, was observed in the liver of rats treated with the highest concentration of troxerutin (TRX-75) compared to the animals of the positive control (DC) group. The significant differences among the treatment groups have been shown by different superscripts (a–c) mentioned on each bar. Data are presented as mean ± SEM (n = 8/group). p < 0.05 was considered statistically significant. Statistical tests: one-way ANOVA followed by Tukey’s post hoc test (applied to normally distributed data) and the Kruskal–Wallis test with Dunn’s correction (applied to non-parametric data). TRX, troxerutin; MET, metformin; NDC, non-diabetic control; DC, diabetic control.

PPARα expression in the liver was significantly (p < 0.05) reduced in the DC rats, and only the highest dose of troxerutin (TRX-75) significantly (p < 0.05) restored PPARα expression (Figure 3). FAS and SREBP-1c expressions were significantly elevated (p < 0.05) in DC rats, but FAS expression was significantly (p < 0.05) downregulated with TRX-75 treatment, while SREBP-1c expression was significantly (p < 0.05) downregulated in both TRX-50- and TRX-75-treated groups (Figure 3).

### 3.7 TNF-α and IL-6 mRNA expressions in the liver, skeletal muscles, and adipose tissue

**FIGURE 4:**
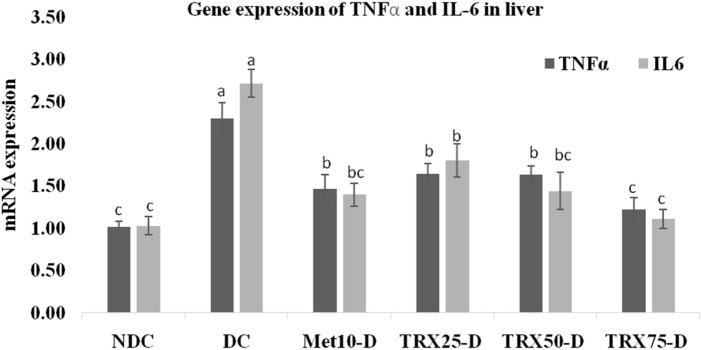
Effects of different concentrations of troxerutin on mRNA gene expressions of TNFα and IL-6 in the liver of rats. Troxerutin significantly (p < 0.05) decreased the expressions of both genes in the liver of rats treated with different concentrations of troxerutin in a dose-dependent manner compared to the rats of the positive control (DC) group. The significant differences among various experimental groups have been represented by different superscripts (a–c) mentioned on each bar. Data are presented as mean ± SEM (n = 8/group). Statistical tests: one-way ANOVA followed by Tukey’s post hoc test (applied to normally distributed data) and the Kruskal–Wallis test with Dunn’s correction (applied to non-parametric data). The statistical significance level was set as p < 0.05. TRX, troxerutin; MET, metformin; NDC, non-diabetic control; DC, diabetic control.

**FIGURE 5:**
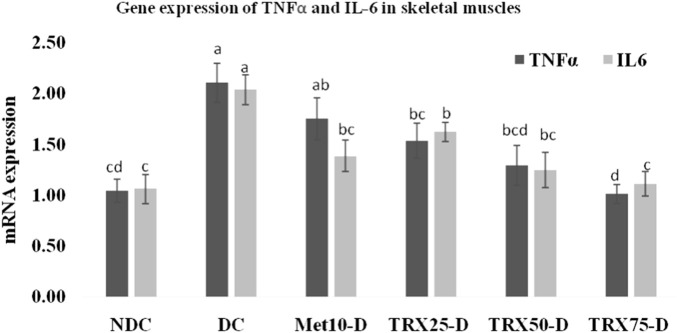
Effects of metformin and different treatments of troxerutin on mRNA gene expressions of TNFα and IL-6 in the skeletal muscles of rats. A significant (p < 0.05) decrease was observed in the gene expressions of both cytokines in the skeletal muscles of treated animals with an increase in the concentration of troxerutin compared to the rats of the positive control (DC) group. The different superscripts (a–d) mentioned on each bar represent the statistical differences among various treatment groups. Statistical tests: one-way ANOVA followed by Tukey’s post hoc test (applied to normally distributed data) and the Kruskal–Wallis test with Dunn’s correction (applied to non-parametric data). Data are presented as mean ± SEM (n = 8/group). p < 0.05 was considered statistically significant. TRX, troxerutin; MET, metformin; NDC, non-diabetic control; DC, diabetic control.

**FIGURE 6:**
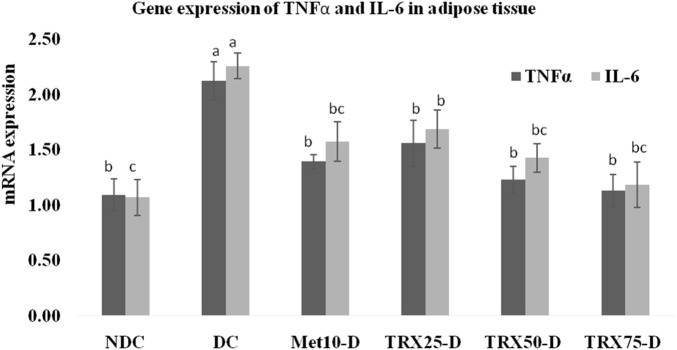
Effects of metformin and different treatment levels of troxerutin on mRNA gene expressions of TNFα and IL-6 in the adipose tissue of rats. A significant (p < 0.05) decrease in the expressions of both the genes was observed in the adipose tissue of rats treated with different concentrations of troxerutin compared to animals of the positive control (DC) group. The different superscripts (a–c) mentioned on each bar represent statistical differences among various experimental groups. Statistical tests: one-way ANOVA followed by Tukey’s post hoc test (applied to normally distributed data) and the Kruskal–Wallis test with Dunn’s correction (applied to non-parametric data). Data are presented as mean ± SEM (n = 8/group). p < 0.05 was considered statistically significant. TRX, troxerutin; MET, metformin; NDC, non-diabetic control; DC, diabetic control.

The expressions of pro-inflammatory cytokines TNF-α and IL-6 were significantly (p < 0.01) upregulated in the liver (Figure 4), skeletal muscles (Figure 5), and adipose tissue (Figure 6) of DC rats compared to the NDC rats. Troxerutin significantly (p < 0.05) downregulated the expression level of both cytokines in all these tissues in a dose-dependent manner, with the greatest effects observed at the highest concentration (TRX-75) compared to the rats of the DC group.

## 4 Discussion

The global burden of DM continues to increase and is expected to reach 11.3% by 2030 and 12.2% by 2045 (Ong et al., 2023). Metformin is currently the only anti-diabetic drug recommended by the American Diabetes Association (Dutta et al., 2023). Meanwhile, various therapeutic agents, including biguanides, thiazolidinediones, metformin, sulfonylurea, and insulin, have been evaluated for glycemic management (Gieroba et al., 2025). Despite their clinical utility, these agents are often accompanied with adverse effects such as hypoglycemia, hepato-toxicity, and cellular apoptosis (Chidambara et al., 2018). Therefore, a safer and more effective treatment regimen with a reduced risk of chronic illness and other side effects is always imperative. Hence, there has been a growing interest in plant-based bioactive components throughout the world, with proficient safety and therapeutic efficacy profile.

Troxerutin, a trihydroxyethylated derivative of the natural bioflavonoid rutin, is well documented for its antioxidant, anti-inflammatory, and anti-hyperlipidemic properties (Geethaet al., 2017; Malinska et al., 2019; Shah et al., 2022). However, these earlier studies primarily focused on its biochemical outcomes, while the present study uniquely integrates a comprehensive analysis of its effects on the expressions of metabolic and inflammatory genes (PPARα, PPARγ, FAS, SREBP-1c, TNF-α, and IL-6) across multiple insulin-responsive body tissues including the liver, skeletal muscles, and adipose tissue. The current molecular-level evaluation distinguishes our work by providing a mechanistic insight into how troxerutin modulates lipid and glucose metabolism pathways compared to metformin.

The recovery of body mass reflects an improved energy utilization and attenuation of muscle wasting commonly observed in uncontrolled diabetes. The concurrent normalization of body weight, fasting glucose levels, and insulin levels indicates a restoration of metabolic homeostasis under troxerutin treatment. The reduction in fasting glucose levels, accompanied with a moderate increase in insulin, implies enhanced peripheral glucose uptake and insulin responsiveness, consistent with upregulation of PPARγ and downregulation of inflammatory cytokines observed in the muscles and adipose tissues. Taken together, these findings demonstrate that troxerutin corrects dyslipidemia and ameliorates whole-body metabolic dysfunction, aligning biochemical outcomes with gene-level modulations of insulin signaling and lipid oxidation pathways.

Dyslipidemia, often associated with T2DM- and HFD-induced obesity, is characterized by elevated TC, TG, LDL, and VLDL levels, along with reduced HDL levels (Kalaivani and Sankaranarayanan, 2019). Troxerutin potentially decreases the levels of TGs and free fatty acids, thus amending the lipid profile in high-fructose- and high-fat-fed rats (Geetha et al., 2017). Similar effects on the lipid profile were reported with metformin monotherapy, in combination with insulin (Sui et al., 2019; Hu et al., 2022; Ismail et al., 2023). Our observations align with previous results, in which troxerutin showed comparable antidiabetic efficacy to that of metformin by significantly (p < 0.05) decreasing the serum TC, LDL, and VLDL levels while (p < 0.001) increasing the HDL level (Shah et al., 2022). We observed that troxerutin treatment significantly decreased the lipid profile (TC, TG, LDL, and VLDL) and increased HDL, thus highlighting its potential to restore lipid balance and reduce the risk of cardiovascular disorders, similar to metformin (Bagnati et al., 2021; Ibrahim et al., 2023; Zhou et al., 2024; Lin et al., 2018). Troxerutin treatment at the highest dose (75 mg/kg/day) also demonstrated a significant improvement in lipid profile parameters including TC, TG, LDL, and VLDL, while increasing the HDL levels. Although these effects exhibited a pattern comparable to that of metformin (10 mg/kg/day), no direct statistical equivalence analysis was conducted between the troxerutin and metformin groups. Therefore, these findings indicate a similar trend in efficacy, and troxerutin has described a comparable directionality rather than complete equivalence to metformin, in modulating lipid metabolism and oxidative stress markers.

We found that troxerutin significantly upregulated (p < 0.05) PPARα and PPARγ (key regulators of lipid metabolism) expressions and downregulated FAS and SREBP-1c (lipogenic genes) expressions, the effects akin to those induced by metformin in the metabolically active tissues such as the liver, skeletal muscles, and adipose tissues. Diabetic dyslipidemia is mitigated by metformin via activation of the AMP-activated protein kinase (AMPK)/ACC/CPT-1 pathway (Dai et al., 2024). Our findings suggest that troxerutin exerts its metabolic benefits through coordinated regulation of the AMPK–PPAR signaling axis, paralleling the mechanism of metformin. Activation of PPARα promotes fatty acid oxidation in hepatic and muscular tissues, while activation of PPARγ enhances insulin sensitivity and glucose uptake in the adipose tissue (Goto et al., 2011). These effects align with the AMPK-mediated energy-sensing pathway targeted by metformin through increased AMP/ATP ratios; AMPK activation suppresses the activity of lipogenic transcription factors such as SREBP-1c and FAS, while stimulating lipid catabolism. The parallel downregulation of these lipogenic genes by troxerutin, together with improvements in oxidative stress and inflammation, supports an AMPK-dependent metabolic reprogramming.

Troxerutin enhances lipid oxidation and glucose utilization primarily through activation of AMPK and its downstream transcriptional targets, PPARα and PPARγ. Activation of AMPK increases fatty acid β-oxidation, suppresses *de novo* lipogenesis by inhibiting the activities of SREBP-1c and FAS, and improves insulin sensitivity by promoting glucose transporter (GLUT4) expression (Malinska et al., 2019). Furthermore, flavonoids such as luteolin and luteolin-7-O-rutinoside, structurally related to troxerutin, have been shown to upregulate PPARγ expression while down-regulating that of SREBP-1c, thereby enhancing insulin responsiveness and glycemic control in STZ-induced diabetic models (Subashraj et al., 2023; Sheh et al., 2022). These mechanisms collectively suggest that troxerutin’s anti-hyperlipidemic and antidiabetic effects involve coordinated AMPK–PPAR signaling, which simultaneously regulates lipid oxidation, glucose homeostasis, and inflammatory balance across hepatic and peripheral tissues. Mechanistically, troxerutin’s antioxidant and anti-inflammatory properties likely preserve mitochondrial integrity and enhance AMPK phosphorylation, thus reinforcing PPAR-driven transcriptional control. This integrated modulation of AMPK, PPARα/γ, and downstream lipid–glucose networks explains the simultaneous normalization of dyslipidemia, insulin resistance, and oxidative stress. In contrast to simply reporting biochemical improvements, this mechanistic interpretation emphasizes the potential of troxerutin as a multitarget metabolic modulator that converges on the same energy-sensing axis as metformin, but with potentially lower cytotoxicity.

Moreover, elevated FAS mRNA expression is related with endothelial dysfunction, diabetes-related complications, and β-cell apoptosis (Kostopoulou et al., 2024). We observed that FAS expression was significantly increased in the adipose tissue of DC rats, but troxerutin treatment significantly (p < 0.05) downregulated its expression, likely by inhibiting the expression of FAS and its ligand, thereby preventing BDE-47-induced renal cytotoxicity in male C57BL/6J mice (Shan et al., 2017). Previously, it has been reported that troxerutin restores insulin sensitivity, reduces lipid contents and oxidative stress in the cardiac tissues, and downregulates SREBP-1c expression in a calorie-rich diet in adult male mice (Geetha et al., 2014). Troxerutin significantly improves hepatic lipid homeostasis by enhancing fatty acid oxidation and suppresses lipogenesis by restoring NAD^+^ depletion-mediated dysfunction in HFD-fed mice (Zhang et al., 2014). This suggests that troxerutin regulates the cholesterol homeostasis level that ultimately reduces the lipid metabolic gene expression by downregulating SREBP-1c expression. PPARα expression in the skeletal muscles and PPARγ expression in adipose tissue were significantly upregulated, indicating an overall improvement in dyslipidemia by modulation of lipid metabolism gene expression (Goto et al., 2011). The biochemical and gene-expression outcomes observed in this study provide mutually reinforcing evidence for multitargeted metabolic effects of troxerutin. The reductions in serum TG, LDL, and VLDL, along with increased HDL, coincide with the enhanced expression of lipid-oxidation regulators (PPARα and PPARγ) and suppression of lipogenic transcription factors (FAS and SREBP-1c). This gene-level modulation likely underlies the biochemical normalization of lipid parameters, indicating that troxerutin promotes a metabolic shift from lipid synthesis toward fatty acid oxidation.

A significant upregulation in the mRNA expressions of pro-inflammatory cytokines TNF-α and IL-6 was observed in the liver, skeletal muscles, and adipose tissues of diabetic rats. However, troxerutin treatment significantly downregulated these cytokines in diabetic rats, thus indicating a potent anti-inflammatory response and a systemic mitigation of inflammation-driven oxidative stress. TNF-α and IL-6 have been associated with β-cell apoptosis and cisplatin-induced oxidative stress in male Wistar rats (Tripathi et al., 2021), STZ-induced diabetic mice (Kotagale et al., 2021), and T2DM-induced renal dysfunction in male Wistar rats (Abdulrahim et al., 2024). Troxerutin has also been previously found to markedly reduce the proliferation of immune cells and reduce the production of pro-inflammatory cytokines such as TNF-α, IL-17, and IL-1 (Jafari-Khataylou et al., 2020). Moreover, troxerutin imparted its anti-inflammatory and antioxidant effects by significantly decreasing the levels of IL-1, IL-17, TNF-α, and IFN-γ in STZ-induced type 1 diabetic C57BL/6 mice (Jafari-Khataylou et al., 2023). Similarly, troxerutin effectively alleviated liver inflammation by mitigating oxidative stress in the liver of BDE-47-treated mice (Zhang et al., 2015). In the same context, these findings strongly support our observations, and these integrated biochemical and molecular responses emphasize troxerutin’s capacity to restore metabolic homeostasis through coordinated transcriptional regulations rather than isolated pathway effects, positioning it as a promising adjunct to metformin for managing diabetic-related inflammation and dyslipidemia. Although several previous studies have established the biochemical efficacy of troxerutin in modulating lipid and glucose metabolism (Geetha et al., 2017; Malinska et al., 2019; Shah et al., 2022), our findings extend this knowledge by demonstrating a coordinated regulation of key metabolic and inflammatory genes across multiple insulin-responsive tissues. This integrative analysis across the liver, skeletal muscles, and adipose tissue provides a more comprehensive understanding of how troxerutin modulates metabolic homeostasis at the molecular level.

Moreover, in addition to its lipid and glycemic regulatory effects, troxerutin markedly improved oxidative balance, as evidenced by reduced MDA levels and enhanced CAT and SOD activities. The elevated oxidative stress in diabetes accelerates lipid peroxidation, impairs insulin signaling, and promotes the production of inflammatory cytokines (Sibony et al., 2024). Troxerutin modulates oxidative stress primarily through activation of the Nrf2–ARE signaling pathway and upregulation of the expressions of endogenous antioxidant enzymes (CAT, SOD, and GPx), leading to decreased lipid peroxidation and restoration of redox homeostasis (Gao et al., 2020). By mitigating these oxidative insults, it has been found that troxerutin restores redox-sensitive transcriptional regulations of PPARα and PPARγ, thereby improving lipid oxidation and glucose uptake. The parallel reduction in MDA and normalization of lipid and inflammatory markers suggest that antioxidant properties of troxerutin act upstream in stabilizing metabolic homeostasis across diverse tissues. This reinforces its potential as a multi-mechanistic botanical metabolite targeting oxidative, inflammatory, and metabolic pathways simultaneously.

The findings of the study provide important insights into the metabolic and molecular effects of troxerutin; however, several limitations should be acknowledged. First, this work was conducted in a preclinical STZ-induced diabetic rat model, which may not fully capture the complexity of type 2 diabetes in humans. Second, only male rats were included, limiting the ability to assess the potential sex-specific responses. Third, the study focused on selected metabolic and inflammatory genes; additional pathways including upstream signaling regulators and broader omics profiles were not examined. Finally, troxerutin pharmacokinetics, long-term safety, and potential interactions with standard antidiabetic drugs were not evaluated. These limitations highlight the need for more mechanistic studies, and well-designed clinical studies are required to validate these preclinical findings and establish troxerutin as a promising botanical compound for comprehensive management of diabetes and its associated metabolic complications.

## 5 Conclusion

This study provides comprehensive evidence that troxerutin exerts multilevel metabolic protection in high-fat-diet-fed streptozotocin-induced diabetic rats. The treatment significantly corrected dyslipidemia by lowering TC, TG, LDL, and VLDL levels while elevating HDL levels, demonstrating a clear improvement in lipid homeostasis. Troxerutin restored hepatic and renal function, as reflected by a marked reduction in AST, ALT, urea, and CRT levels, and it improved oxidative balance by decreasing MDA levels and enhancing catalase activity. These findings indicate that troxerutin mitigates metabolic derangements and prevents secondary organ damage commonly associated with uncontrolled diabetes. At the molecular level, troxerutin consistently modulated key metabolic and inflammatory markers across multiple insulin-responsive tissues. Upregulation of the expressions of PPARα and PPARγ, combined with downregulation of FAS, SREBP-1c, TNF-α, and IL-6, points toward a coordinated enhancement of lipid oxidation, reduction in lipogenesis, and attenuation of tissue-level inflammatory stress. This cross-tissue transcriptional regulation emphasizes the potential of troxerutin as a broad-spectrum metabolic modulator rather than a single-pathway agent.

The direction of these effects parallels the metabolic improvements observed with metformin, suggesting that troxerutin exerts complementary benefits through a distinct phytochemical mechanism. Compared with earlier studies that focused on isolated biochemical endpoints, this work expands our current knowledge by elucidating a cross-tissue, gene-level mechanism that explains the consistent metabolic improvements observed. Taken together, these results indicate that troxerutin meaningfully improves glycemic control, lipid management, antioxidant status, and inflammatory signaling. Its dose-dependent responses and multigene regulatory actions strengthen its potential as a natural adjunct or alternative to conventional antidiabetic therapies.

## Data Availability

The original contributions presented in the study are included in the article/supplementary material; further inquiries can be directed to the corresponding authors.
